# Successful rescue of the ruptured chronic B dissecting aneurysm after endovascular stent-graft with supraclavicular carotid artery graft cannulation

**DOI:** 10.1186/1749-8090-10-S1-A40

**Published:** 2015-12-16

**Authors:** Chul Hyun Park, Kuk Hui Son, Chang-Hyu Choi, Jae-Ik Lee, Kun Woo Kim, Ji Sung Kim, So Young Lee, Kook Yang Park

**Affiliations:** 1 Department of Thoracic and Cardiovascular Surgery, Gachon University Gil Medical Center, Incheon, 405-760, Republic of Korea

## Background/Introduction

The thoracic endovascular repair (TEVAR) has been developed to cover the primary entry in the chronic dissecting aneurysm. The TEVAR is useful in the life-threatening condition of type B dissection with rupture or malperfusion.

## Aims/Objectives

After a failed endovascular repair, complications are rare, but fatal, thus requires a surgical conversion. We report a case in which the prepared carotid cannulation saved the ruptured chronic dissecting aneurysm.

## Method

A 44-year-old patient underwent the TEVAR with chimney technique of left subclavian artery in a life-threatening situation of type B chronic dissecting aneurysm with rupture. A follow-up CT scan showed the proximal type I endoleak with high pressurized aneurysm 5 days later. The graft explanation and open repair were performed emergently. Via a left supraclavicular incision, the end-to side graft cannulation in left carotid artery and the carotid-to-subclavian bypass were performed for a proximal antegrade perfusion and a salvage of left subclavian artery. The femoro-femoral bypass was prepared in the left inguinal area. As soon as the left thoracotomy was performed, the stented descending aorta ruptured. CPB was instituted with the left common carotid artery and the femoro-femoral bypass. The operative fields were secured with digital compression and a cross-clamping on the mid-thoracic aorta. Fortunately, the proximal perfusion was achieved with digital compression, balloon occlusion and aortic clamping on the distal arch after the thoracic stent-graft and the chimney graft were explanted. The ruptured dissecting aneurysm was reconstructed using an interposition of graft.

## Results

The postoperative course was uneventful without any mental change or spinal injury. The survived patient was in a good condition during the follow-up of 7 months.

## Discussion/Conclusion

The additional cannulation of end-to side graft in left carotid artery would be a life-saving tool in the a complicated condition like impending rupture of descending thoracic aortic aneurysm.

## Consent

Written informed consent was obtained from the patient for publication of this abstract and any accompanying images. A copy of the written consent is available for review by the Editor of this journal.

